# Insights on safety and efficacy of renal artery denervation for uncontrolled-resistant hypertension in a high risk population with chronic kidney disease: first Italian real-world experience

**DOI:** 10.1007/s40620-021-00966-7

**Published:** 2021-01-22

**Authors:** Federico Marin, Simone Fezzi, Alessia Gambaro, Francesco Ederle, Gianluca Castaldi, Maddalena Widmann, Concetta Gangemi, Valeria Ferrero, Gabriele Pesarini, Michele Pighi, Flavio L. Ribichini

**Affiliations:** 1 Division of Cardiology, Department of Medicine, University of Verona, Piazzale Aristide Stefani 1, 37126 Verona, Italy; 2 Division of Nephrology, Department of Medicine, University of Verona, Verona, Italy

**Keywords:** Renal artery denervation, Uncontrolled resistant hypertension, Chronic kidney disease, End-stage kidney disease

## Abstract

**Aims:**

To evaluate the safety and efficacy of catheter-based radiofrequency renal sympathetic denervation (RSD) in a daily practice population of patients with uncontrolled resistant hypertension, on top of medical therapy.

**Methods:**

Consecutive unselected patients with uncontrolled resistant hypertension undergoing RSD were enrolled. Office and ambulatory blood pressure (BP) measurements were collected at baseline and 3, 6 and 12 months after RSD. Efficacy was assessed even in patients with an estimated glomerular filtration rate (eGFR) below 45 mL/min/1.73 m^2^. Patients were defined as responders if systolic BP decreased by at least 5 mmHg at ambulatory BP or by 10 mmHg at office BP at their last follow-up visit.

**Results:**

Forty patients with multiple comorbidities underwent RSD from 2012 to 2019. Baseline office and ambulatory BP was 159.0/84.9 ± 26.2/14.9 mmHg and 155.2/86.5 ± 20.9/14.0 mmHg, respectively. At 12-month follow up a significant reduction in office and ambulatory systolic BP, respectively by − 19.7 ± 27.1 mmHg and by − 13.9 ± 23.6 mmHg, was observed. BP reduction at 12-month follow-up among patients with eGFR < 45 mL/min was similar to that obtained in patients with higher eGFR. Twenty-nine patients (74.4%) were responders. Combined hypertension, higher ambulatory systolic BP and lower E/E’ at baseline emerged as predictors of successful RSD at univariate analysis. No major complications were observed and renal function (was stable up to 12 months), even in patients with the lowest eGFR values at baseline.

**Conclusion:**

RSD is safe and feasible in patients with uncontrolled resistant hypertension on top of medical therapy, even in a high-risk CKD population with multiple comorbidities, with a significant reduction in systolic BP and a trend towards a reduction in diastolic BP lasting up to 12 months.

**Graphic abstract:**

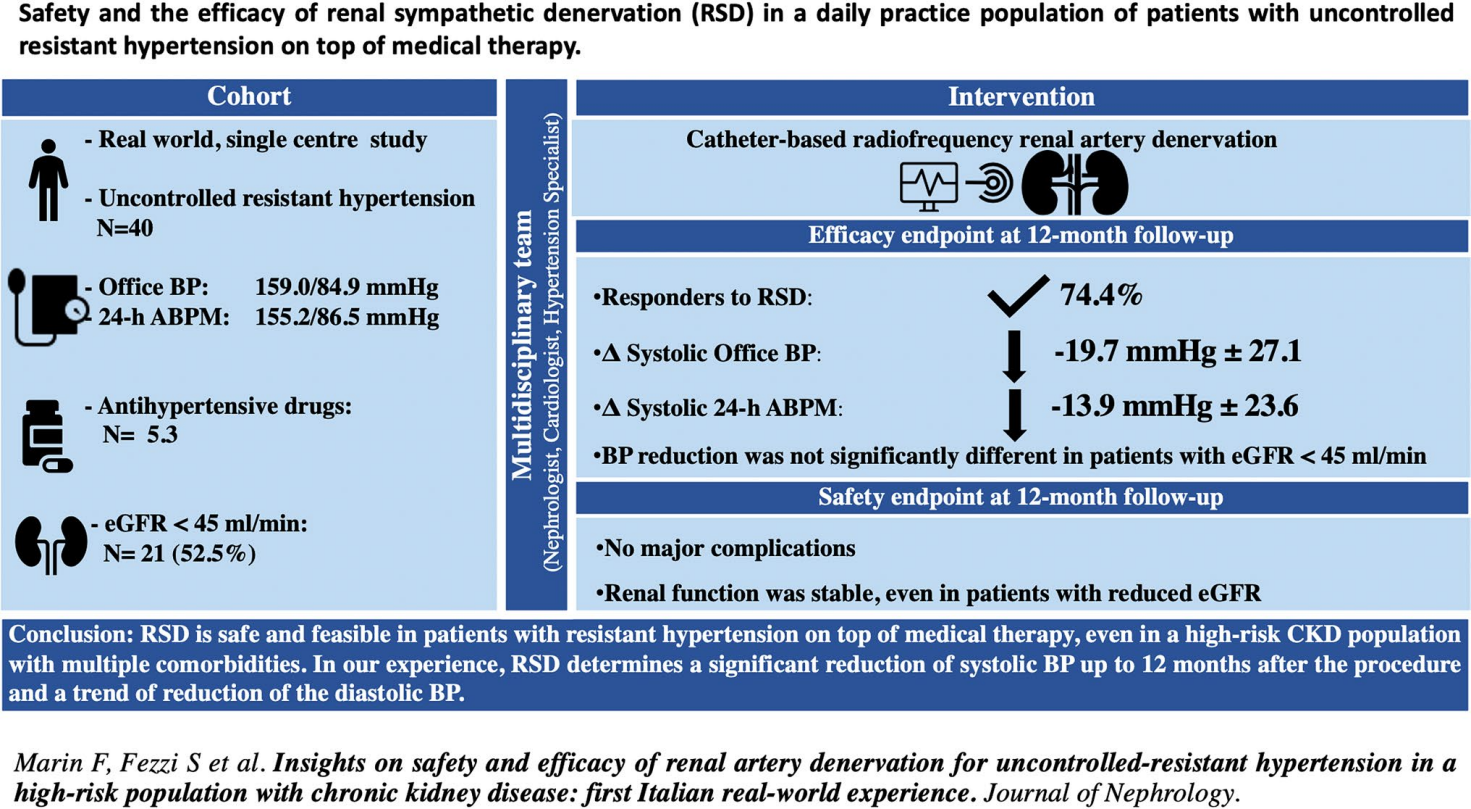

**Supplementary Information:**

The online version contains supplementary material available at 10.1007/s40620-021-00966-7.

## Introduction

Uncontrolled resistant hypertension is defined as systolic blood pressure of 140 mmHg or higher despite the adherence to at least three antihypertensive medications, including a diuretic, at maximally tolerated doses [1]. Although the prevalence of uncontrolled resistant hypertension is difficult to estimate, it ranges from 9 to 15% in adult populations [2, 3], rising up to 21.1% in patients with chronic kidney disease (CKD) [4]. Resistant hypertension is associated with poor prognosis and increases the risk of major cardiovascular events [5, 6]. Catheter-based radiofrequency renal sympathetic denervation (RSD) has emerged as a therapeutic option [7, 8], and initial studies, such as the Symplicity HTN-1 and HTN-2 revealed a significant reduction in blood pressure (BP) [9, 10]. The results were not confirmed however by the following double blind, randomized, Symplicity HTN-3 trial in which the treatment was compared to a sham procedure [11]. Several interpretations have been put forth to analyze the possible causes of the negative results of HTN-3 [12]. Based on some anatomical observations related to the innervation of the renal arteries, a different pattern of radiofrequency delivery was hypothesized, which together with the release of a new catheter with four circumferentially placed electrodes, rekindled interest in the technique. Several preclinical studies followed, which demonstrated a significant correlation between the modality of the RSD technique, the release of norepinephrine from the kidney, and the reduction in BP [13, 14]. To overcome the limitations of the Symplicity HTN studies, a second generation of studies, named Spyral HTN trials, were initiated to re-evaluate the efficacy of RSD by adopting both improved study methodology and a homogeneous population, as well as by using the new tetrapolar catheter and extending the treatment to the distal branches of the renal artery [15]. The results of these proof of concept studies were encouraging, showing the biological signal that RSD decreases BP [16–18]. However, few data are available regarding daily practice, in particular for patients with CKD who were systematically excluded from the cited trials.

This article reports the prospective clinical experience at our center and aims to evaluate the safety and the efficacy of RSD in unselected patients with uncontrolled resistant hypertension and multiple comorbidities, especially CKD, in a real-world clinical setting.

## Methods

All patients were referred to our Institution because of uncontrolled resistant hypertension. They were screened and eligibility for RSD was discussed among members of a multidisciplinary team composed of clinical and interventional cardiologists, nephrologists and hypertension specialists.

Eligible patients had office systolic BP above 140 mmHg while on at least three antihypertensive medications from complementary classes, including a diuretic, all of which at the maximum tolerated doses.

Exclusion criteria were secondary forms of hypertension other than those related to CKD, significant valvular heart disease, recent acute cardiovascular events (acute myocardial infarction, stroke or pulmonary embolism in the last three months), and hemodynamically significant renal artery stenosis (diameter stenosis > 70%) with demonstration of a significant (> 20 mmHg) trans-lesional pressure gradient [19]. Furthermore, patients under 18 years of age and pregnant women were excluded. Nonadherence to medical therapy was ruled out in all patients during hospital admission when medical therapy was confirmed by the investigators. In a small subgroup of patients (*n* = 3) therapy adherence was confirmed by urine samples. During follow-up medical adherence was assessed by direct questioning. In order to rule out a diagnosis of white coat hypertension, all patients were screened based on their home blood pressure diary, and in 24 patients based on basal 24-h ambulatory blood pressure monitoring (ABPM).

The enrollment protocol included clinical evaluation, imaging of the renal arteries, and blood sample collection. Office blood pressure (OBP) was measured as the mean of three recordings in the sitting position after a 5-min rest by mercury sphygmomanometer. 24-h ABPM was recorded using “GE-TONOPORT V” automated oscillometric devices; measurement intervals were 15 min during daytime and 30 min during night-time; a minimum of 70% of valid measurements was required. The protocol was approved by our Ethics Committee (CESC-2361) and by the Department of Health of our Region. All patients signed informed consent and were enrolled in the Global Symplicity Registry (GSR) [20].

Each RSD procedure was performed by an expert interventional cardiologist. The procedure involves endovascular access via the femoral artery with advancement of a catheter-mounted device into the renal artery through a 6Fr guiding catheter and a floppy 0.014″ coronary wire. Local anesthesia and sedatives were administered to relieve patient discomfort. A renal angiogram was obtained to confirm suitability of the target vessel and was repeated at the end of the procedure. Until 2014, eligible patients underwent bilateral RSD using the unipolar catheter (Symplicity Flex™, Medtronic), while the tetrapolar catheter (Symplicity Spyral™, Medtronic) was used thereafter. The automated algorithms in the generator box (Symplicity G2™, Medtronic) continuously monitored temperature and impedance at the point of ablation to optimize the delivery of the radiofrequency energy.

Follow-up visits consisting of clinical evaluation, accurate OBP measurements, assessment of medication intake by direct questioning, blood sample collection and ABPM were recommended 3, 6 and 12 months after the procedure. Renal artery imaging during follow-up was performed only if clinically indicated.

Safety endpoints included; the absence of any device-related major complication, defined as any peri-procedural major vascular complication including renal artery perforation or dissection, any significant embolic event resulting in target organ damage, major bleeding as defined by the BARC (Bleeding Academic Research Consortium) classification [21], end-stage renal disease (ESRD), stroke, acute myocardial infarction, and any cause of death within 1 month of the procedure. Any other complication related to the procedure was classified as minor.

Efficacy endpoint was determined by the interindividual change in OBP and ABPM from baseline to twelve months after the procedure, with interim analysis at three and six months. Variations from baseline of peripheral pulse pressure (p-PP), heart rate (HR), serum creatinine, and number of medications were also assessed.

A secondary pre-specified analysis was conducted to evaluate the efficacy of RSD in patients with an eGFR below 45 mL/min/1.73 m^2^ compared to the group with eGFR ≥ 45 mL/min/1.73 m^2^, since they had been excluded per-protocol from the Simplicity HTN-1, HTN-2 and HTN-3 trials.

A tertiary analysis included a comparison between “responders” and “non-responders” in terms of BP drop after the procedure. According to the literature, if ABPM had not been performed, patients were defined as responders if systolic BP decreased by at least 5 mmHg at ambulatory BP or by 10 mmHg at office BP at their last available follow-up visit [22, 23].

Statistical methods: continuous variables are presented as mean and standard deviation. Categorical data are reported as number and percentage. Comparisons between continuous variables were performed using the paired sample *t* test. Comparisons between categorical variables were performed using Fisher’s exact test. Analysis of Covariance was employed to adjust for baseline blood pressure measurements. Binary logistic regression was applied to identify predictors of successful RSD, while multivariate analysis was not conducted due to the small sample size. A probability value of *p* ≤ 0.05 was considered statistically significant. All statistical analyses were performed using SPSS 26.0 (IBM Inc., USA).

## Results

### Baseline characteristics

From 2013 to 2019, forty consecutive patients underwent RSD at our center and were included in the present analysis. Table 1 lists the baseline characteristics and procedural details of the population. Mean age was 60.6 ± 14.3 years and 77.5% were male. Baseline OBP was 159.0/84.9 ± 26.2/14.9 mmHg, ABPM was available for 24 patients (60%) and was 155.2/86.5 ± 20.9/14.0 mmHg. At baseline, patients were prescribed 5.3 ± 1.1 antihypertensive drug classes. One patient out of three was on a mineralocorticoid receptor antagonist. The most prevalent comorbidity was CKD (*n* = 30, 75%), including 19 patients in class III CKD, 7 in class IV and 4 on hemodialysis at study entry. Mean eGFR was 46.7 ± 27.36 mL/min/1.73 m^2^. Other comorbidities were diabetes mellitus (DM) (*n* = 22, 55%) and peripheral artery disease (PAD) (*n* = 13, 32.5%). Echocardiographic data were available for 29 patients. Mean left ventricle ejection fraction was 56% ± 9.2 and the mean thickness of the interventricular septum was markedly increased (14 ± 2.9 mm). Apart from the severity of CKD, baseline characteristics were similar between the two groups (eGFR < 45 mL/min and eGFR ≥ 45 mL/min).

**Table 1:** Baseline characteristics and procedural details

	Baseline		Overall *n* = 40 (100%)	eGFR < 45 mL/min *n* = 21 (52.5%)	eGFR ≥ 45 mL/min *n* = 19 (47.5%)	*p* value
Clinical characteristics	Age (years)		60.6 ± 14.3	59.67 ± 17.05	61.89 ± 10.89	*p* = 0.622
	Male gender		31; 77.5%	18; 85.7%	13; 68.4%	*p* = 0.177
	BMI (kg/m^2^)		30.6 ± 5.3	29.5 ± 5.7	31.8 ± 5.0	*p* = 0.186
	Race: Caucasian		36; 90.0%	18; 85.7%	18; 94.7%	*p* = 0.342
	Smoking		19; 47.5%	8; 38.1%	11; 57.9%	*p* = 0.342
	eGFR (mL/min)		46.7 ± 27.4 [3.4–105]	25.9 ± 11.5	69.6 ± 20.5	***p < 0.001***
	CKD		30; 75%	21; 100%	9; 47.4%	***p < 0.001***
	Stage III CKD		19; 47.5%	10; 47.6%	9; 47.4%	*p* = 1.000
	Stage IV CKD		7; 17.5%	7; 33%	–	***p = 0.009***
	Stage V CKD		4; 10%	4; 19%	–	*p* = 0.108
	Diabetes, Type 2		22; 55%	13; 61.9%	9; 47.4%	*p* = 0.525
	Isolated systolic hypertension		24; 60%	11; 52.4%	13; 68.4%	*p* = 0.349
	Family history of hypertension		18; 45%	12; 57.1%	6; 31.6%	*p* = 0.125
	PAD		13; 32.5%	7; 33.3%	6; 31.6%	*p* = 1.000
	History of cardiac disease		12; 30%	4; 19%	8; 42.1%	*p* = 0.170
	COPD		8; 20%	5; 23.8%	3; 15.8%	*p* = 0.698
	Obstructive sleep apnea		5; 12.5%	2; 9.5%	3; 15.8%	*p* = 0.654
	Antihypertensive medications		5.3 ± 1.1	5.5 ± 0.9	5.1 ± 1.2	*p* = 0.305
Blood pressure	Office-SBP (mmHg)		159.0 ± 26.2	157.7 ± 22.4	160.7 ± 30.4	*p* = 0.716
	Office-DBP (mmHg)		84.9 ± 14.9	89.1 ± 15.5	80.4 ± 13.2	*p* = 0.066
	24-h SBP (mmHg)	*n* = 24	155.2 ± 20.9	157.1 ± 18.0	153.3 ± 24.1	*p* = 0.670
	24-h DBP (mmHg)		86.5 ± 14.0	89.8 ± 16.3	83.3 ± 11.1	*p* = 0.272
	Heart rate (bpm)		68.5 ± 10.6	70.2 ± 10.8	66.9 ± 10.3	*p* = 0.332
Echocardriogram	LV EF (%)	*n* = 29	56 ± 9.2	55.7 ± 8.8	56.4 ± 10.0	*p* = 0.845
	LV VTD (mL/m^2^)	*n* = 26	70.2 ± 24.0	71.1± 29.1	69.0 ± 15.8	*p* = 0.833
	IVS (mm)	*n* = 25	14 ± 2.9	13.9 ± 2.7	14.2 ± 3.2	*p* = 0.832
	LA (mL/m^2^)	*n* = 27	44.7 ± 10.9	38.4 ±—15.3	46.2 ± 8.2	*p* = 0.448
	*E*/*E*′	*n* = 21	12 ± 3.4	11.7 ± 2.9	12.3 ± 3.9	*p* = 0.706
	*e*/*a*	*n* = 22	1.2 ± 0.7	1.1 ± 0.6	1.3 ± 0.7	*p* = 0.656
Procedural details	Ablation points		36.2 ± 16.0	32.5 ± 15.6	40.2 ± 15.0	*p* = 0.131
	Flex catheter		6; 15%	4; 19%	2; 10.5	*p* = 0.664
	Spyral catheter		34; 85%	17; 81%	17; 89.5%	*p* = 0.664
	Treatment time (min)		53.0 ± 14.0	54.2 ± 11.2	50.8 ± 16.2	*p* = 0.305
	Radioscopy duration (min)		12.0 ± 5.0	11.5 ± 4.4	13.1 ± 4.9	*p* = 0.441
	Contrast volume (mL)		71.9 ± 39.5	71.4 ± 34.6	72.4 ± 45.5	*p* = 0.938
	Major complication		0	0	0	–
	Minor complication		6; 15%	4; 19%	5; 10.5%	*p* = 0.381
	Transient increment of creatinine		4; 10%	3; 14.3%	1; 5.3%	*p* = 0.342
	Femoral pseudoaneurysm		2; 5%	1; 4.8%	1; 5.3%	*p* = 1.000

*BMI* Body mass index, *CKD* chronic kidney disease, *COPD* chronic obstructive pulmonary disease, *DBP* diastolic arterial pressure, *eGFR* estimated glomerular filtration rate, *IVS* interventricular septum, *LA* left atrial, *LV EDV* left ventricle end diastolic volume, *LV EF* left ventricle ejection fraction, *PAD* peripheral artery disease

### Procedural details

Until 2014, six patients (15%) were treated with the unipolar catheter (Flex group), while between 2015 and 2019, thirty-four (85%) underwent RSD using the tetrapolar catheter (Spyral group). Bilateral RSD was performed in 38 (95%) patients since one patient had a chronic total occlusion of the left renal artery and one had a single kidney after surgical right nephrectomy [24]. Comparisons between the Flex and Spyral group are reported in Table S.1 and reflect the different instructions for use: the mean number of total ablation points was significantly higher in the Spyral group (40.56 ± 13.03 vs 11.33 ± 3.45; *p* < 0.001) and the target ablation points in the Flex group were limited to the main artery alone, whereas treatment was delivered to the main artery and extended to the distal branches as well in the Spyral group. Furthermore, as a possible consequence of the operator’s learning curve, the amount of contrast medium administered was significantly lower in the Spyral group (61.00 ± 17.79 mL vs 133.33 ± 69.4 mL; *p* < 0.001). No other significant differences were noted in terms of procedural duration or radioscopy time.

### Safety endpoints

**Fig. 1:**
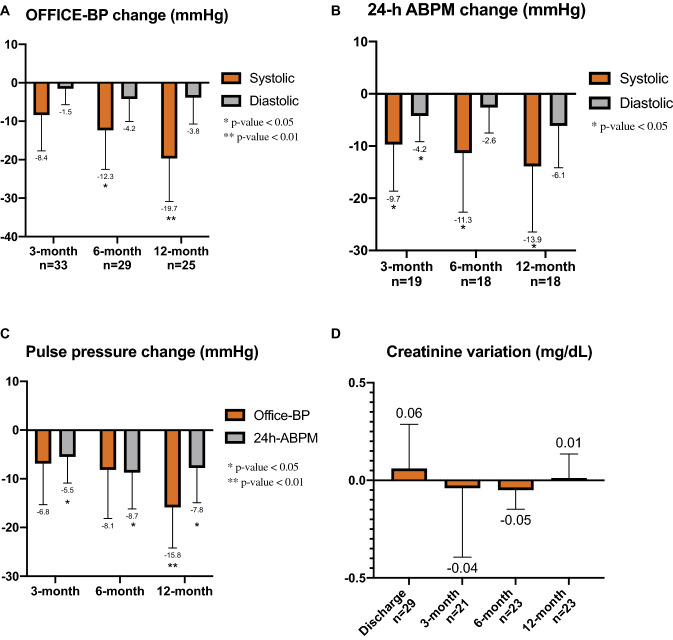
Interindividual change (mean, 95% CI) in **a** office blood pressure, **b** 24-h ambulatory blood pressure, **c** peripheral pulse pressure and **d** serum creatinine at follow-up

None of the 40 patients experienced any major complications as defined by the protocol. Three patients (13.4%) in the eGFR < 45 mL/min group and one patient (5.3%) in the other group had a transient increase in serum creatinine, defined by an absolute increase of about 0.3 mg/dL from basal values, that normalized after hydration therapy for 48 h. Two patients with uncontrolled BP (220/120 mmHg) values developed femoral pseudo-aneurysm despite all the precautions taken to avoid this complication and therefore required thrombin embolization. No significant interindividual differences in serum creatinine were observed at discharge or during follow-up, as shown in Fig. 1d. No significant difference in terms of creatinine variation during follow-up was noticed between groups as shown in Table 2. Two patients with ESRD and uncontrolled resistant hypertension underwent successful kidney transplantation without native kidney nephrectomy after improving BP control post-RSD, and both patients maintained good BP control after transplantation.

**Table 2:** Blood pressure reduction and creatinine variation between groups

		eGFR < 45 mL/min *n* = 21 (52.5%)		eGFR ≥ 45 mL/min *n* = 19 (47.5%)		*p* value
Efficacy primary endpoint						
Office BP reduction (SBP/DBP mmHg)	12-month FU	*n* = 12	− 19.42/− 9.50 ± 31.57/17.08	*n* = 13	− 19.92/1.38 ± 23.59/14.99	*p* = 0.998/*p* = 0.762
24-h ABPM reduction (mmHg)	12-month FU	*n* = 8	− 18.00/− 3.87 ± 23.4/11.93	*n* = 8	− 9.75/− 0.25 ± 24.58/14.27	*p* = 0.407/*p* = 0.764
Creatinine variation	3-month FU	*n* = 10	− 0.14 ± 0.27	*n* = 11	0.06 ± 0.18	*p* = 0.059
Creatinine variation	6-month FU	*n* = 13	− 0.10 ± 0.40	*n* = 10	0.01 ± 0.12	*p* = 0.374
Creatinine variation	12-month FU	*n* = 12	− 0.09 ± 0.75	*n* = 11	0.11 ± 0.28	*p* = 0.410

### Efficacy endpoints

Paired OBP measurements were available for 33 (83% of the eligible samples) patients at 3-month, 29 (81%) at 6-month, and 25 (76%) at 12-month follow-up, and paired data on ABPM were available for 19 (48%) patients at 3-month, 18 (50%) at 6-month, and 16 (48%) at 12-month follow-up. All patients underwent at least one follow-up visit. Two patients died from non-cardiac causes 10 and 11 months after the procedure, respectively. BP follow-up data collection was limited since several patients were referred and then followed-up at other institutions.

**Fig. 2:**
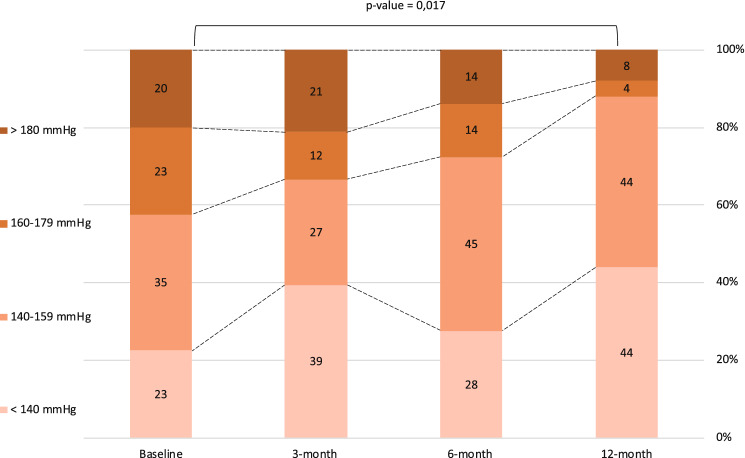
Change in office-SBP during follow-up

The mean change in OBP from baseline is shown in Fig. 1a. At 3-month follow-up, office systolic BP decreased by − 8.4 ± 26.4 mmHg (*p* = 0.078) and office diastolic BP by − 1.5 ± 11.8 mmHg (*p* = 0.467). At 6-month follow-up, office systolic BP decreased by − 12.4 ± 26.7 mmHg (*p* = 0.019) and office diastolic BP by − 4.2 ± 15.4 mmHg (*p* = 0.152). At 12-month follow-up office systolic BP decreased by − 19.7 ± 27.1 mmHg (*p* = 0.001) and office diastolic BP decreased by − 3.8 ± 16.6 mmHg (*p* = 0.260). The proportion of patients that achieved target BP after RSD, defined as an office systolic BP < 140 mmHg, was 39%, 28% and 44% respectively at 3-month, 6-month and 12-month follow-up (Fig. 2) BP reduction in the group of patients treated with the Flex catheter (*n* = 6) compared to the Spyral catheter (*n* = 34) is reported in Table S.2. The mean individual change in ABPM from baseline is shown in Fig. 1b. At 3-month follow-up, 24-h systolic BP decreased by − 9.7 ± 18.6 mmHg (*p* = 0.036) and 24-h diastolic BP by − 4.2 ± 10.3 mmHg (*p* = 0.091). At 6-month follow-up, 24-h systolic BP decreased by − 11.3 ± 22.8 mmHg (*p* = 0.050) and 24-h diastolic BP by − 2.6 ± 9.8 mmHg (*p* = 0.275). At 12-month follow-up, 24-h systolic BP decreased by − 13.9 ± 23.6 mmHg (*p* = 0.033) and 24-h diastolic BP decreased by − 6.1 ± 15.1 mmHg (*p* = 0.124). The proportion of patients that achieved target 24-h systolic BP at 12-month follow-up was 39%. The effect of RSD on p-PP is shown in Fig. 1c. At baseline, mean ambulatory p-PP was 68.7 ± 16.6 mmHg, while office p-PP was 72.0 ± 21.5 mmHg. A significant reduction in ambulatory p-PP was seen at 3-month follow-up (− 5.47 ± 11.18 mmHg, *p* = 0.047), at 6-month follow-up (− 8.72 ± 14.97 mmHg, *p* = 0.024) and at 12-month follow-up (− 7.75 ± 13.45 mmHg, *p* = 0.036). The reduction in office p-PP was statistically significant at 12-month follow-up (− 15.84 ± 20.27 mmHg, *p* = 0.001). During follow-up, non-significant changes were observed in terms of HR and number of medications.

A secondary analysis compared the primary end point in the group with eGFR < 45 mL/min, as reported in Table 2. At 12-month follow-up no significant difference was noted in terms of reductions in office BP, even after adjustment for baseline measurements (eGFR < 45 mL/min: − 19.42/− 9.50 ± 31.57/17.08 mmHg vs eGFR ≥ 45 mL/min: − 19.92/1.38 ± 23.59/14.99 mmHg; *p* = 0.998/*p* = 0.762). At 12-month follow-up no significant difference was noted in terms of reductions in 24-h BP between groups, adjusted for baseline measurements (eGFR < 45 mL/min: − 18.00/− 3.87 ± 23.4/11.93 mmHg vs eGFR ≥ 45 mL/min: − 9.75/− 0.25 ± 24.58/14.27 mmHg; *p* = 0.407/*p* = 0.764). No interindividual changes were noted in terms of HR, serum creatinine and number of medications during follow-up between groups.

### Subgroup analysis of responders

On the basis of the chosen definition, twenty-nine patients (74.4%) were “responders” at their last available follow-up, as reported in Table 3. In the responder group, ABPM and OBP at baseline were significantly higher compared to the non-responder group (ABPM: 162.35/90.82 ± 19.57/12.3 mmHg vs 137.86/76.1 ± 12.6/13.2 mmHg; OBP: 164.93/88.14 ± 27.27/15 mmHg vs 146.2/76.1 ± 14.03/12.06 mmHg). Moreover, baseline HR was higher in the responder group (70.07 ± 11.11 vs 63.3 ± 7.01; *p* = 0.034). In the non-responder group prevalence of isolated systolic hypertension (ISH) was significantly higher (90% vs 48.3; *p* = 0.028) as was the *E*/*E*′ ratio (10.54 ± 2.5 vs 14.43 ± 3.55; *p* = 0.029) compared to responders. Non-significant differences were found in the procedural characteristics. No significant differences were observed in the average number of medications at baseline and during follow-up between responders and non-responders.In the responder-group, the number of medications at 12-month follow-up was significantly lower compared to the number of medications before RSD was performed (5.14 ± 1.04 vs. 5.41 ± 1.01; *p* = 0.01).

**Table 3:** Comparison between responders and non-responders

Baseline	Overall *n* = 39^§^	Responder *n* = 29 (74.4%)	Non responder *n* = 10 (34.5%)	*p* value
Clinical characteristics				
Age (years)		59.9 ± 14.01	64.8 ± 15.04	*p* = 0.380
Male gender		22; 75.9%	8; 80%	*p* = 1.000
BMI (kg/m^2^)		30.53 ± 5.73	31.19 ± 4.75	*p* = 0.723
Race: Caucasian		27; 93.1%	9; 90%	*p* = 1.000
Smoking		13; 44.8%	5; 50%	*p* = 1.000
eGFR (ml/min)		48.31 ± 29.49	41.3 ± 22.2	*p* = 0.440
Chronic kidney disease		20; 69%	9; 90%	*p* = 0.402
Stage III CKD		12;41.4%	6; 60%	*p* = 0.465
Stage IV CKD		4; 13.8%	3; 30%	*p* = 0.344
Stage V CKD		4; 13.8%	0	*p* = 0.556
Diabetes, Type 2		15; 51.7%	6; 60%	*p* = 0.726
Isolated systolic hypertension		14; 48.3%	9; 90%	*p* = 0.028
Family history of hypertension		15; 51.7%	2; 20%	*p* = 0.140
Peripheral artery disease		8; 27.6%	5; 50%	*p* = 0.253
History of cardiac disease		7; 24.1%	5; 50%	*p* = 0.232
COPD		7; 24.1%	1; 10%	*p* = 0.653
Obstructive sleep apnea		3; 10.3%	2; 20%	*p* = 0.587
Antihypertensive medications (baseline)		5.41 ± 1.01*	5.22 ± 1.21	*p* = 0.745
Antihypertensive medications (3–6 months)		5.30 ± 1.02	5.00 ± 1.31	*p* = 0.304
Antihypertensive medications (12 months)		5.14 ± 1.04*	4.86 ± 1.07	*p* = 0.558
Blood pressure				
Office-SBP (mmHg)		164.93 ± 27.27	146.2 ± 14.03	*p* = 0.002
Office-DBP (mmHg)		88.14 ± 15	76.1 ± 12.06	*p* = 0.029
Office Pulse Pressure (mmHg)		76.8 ± 27.7	70.1 ± 17.9	*p* = 0.481
24-h SBP (mmHg)	*n* = 24	162.35 ± 19.57	137.86 ± 12.6	*p* = 0.009
24-h DBP (mmHg)		90.82 ± 12.3	76.14 ± 13.2	*p* = 0.019
24-ABPM Pulse Pressure(mmHg)		71.5 ± 18.1	61.7 ± 9.9	*p* = 0.105
Heart rate (bpm)		70.07 ± 11.11	63.3 ± 7.01	*p* = 0.034
Echocardiogram				
LV EF (%)	*n* = 28	58.26 ± 7.61	52.11 ± 11.43	*p* = 0.169
LV VTD (mL/mq)	*n* = 25	73.71 ± 24.17	60.38 ± 22.88	*p* = 0.203
Interventricular septum (mm)	*n* = 24	13.76 ± 2.63	14.57 ± 3.69	*p* = 0.612
Left atrial volume (mL/mq)	*n* = 26	45.43 ± 11.12	43.59 ± 11.72	*p* = 0.703
*E*/*E*′	*n* = 20	10.54 ± 2.5	14.43 ± 3.55	*p* = 0.029
*e*/*a*	*n* = 21	1.08 ± 0.56	1.36 ± 0.82	*p* = 0.401
Procedural details				
Spyral™ catheter		25; 86.2%	8; 80%	*p* = 0.636
Ablation points (right-left)		36.41 ± 15.79	34.4 ± 17.9	*p* = 0.757
Main vessel treatment only		4; 13.7%	2; 20%	*p* = 0.636
Main vessel + any branch treatment		25; 86.2%	8; 80%	*p* = 0.639
Treatment time (min)		53.34 ± 12.02	50.8 ± 18.9	*p* = 0.697
Radioscopy duration (min)		12.55 ± 4.7	11.67 ± 4.87	*p* = 0.639
Contrast volume (mL)		71 ± 29.55	77 ± 63.08	*p* = 0.078
Major procedural complication		0	0	–
Minor procedural complication		4; 13.8%	2;20%	*p* = 0.636
Transient increment of creatinine		2; 6.9%	2; 20%	*p* = 0.267
Groin hematoma		2; 6.9%	0	*p* = 1.000

*BMI* Body mass index, *COPD* chronic obstructive pulmonary disease, *DBP* diastolic arterial pressure, *eGFR* estimated glomerular filtration rate, *IVS* interventricular septum, *LA* left atrial, *LV EDV* left ventricle end diastolic volume, *LV EF* left ventricle ejection fraction, *PAD* peripheral artery disease

*p = 0.01

^§^One patient lost to follow-up

Univariate analysis demonstrated that higher 24 h systolic BP (OR 1.13; 95% CI 1.01–1.25; *p* = 0.028), combined with hypertension (odds ratio [OR] 9.64; 95% confidence interval [CI] 1.08–86.21; *p* = 0.043), and lower *E*/*E*′ (OR 0.61; 95% CI 0.39–0.98; *p* = 0.039) were predictors of successful RSD. Multivariate analysis was not performed due the small sample size.

## Discussion

We report on the safety and efficacy of radiofrequency-based RSD treatment applied in a real-life, unselected population of patients with uncontrolled resistant hypertension treated with the maximum tolerated anti-hypertensive medical therapy as advocated by the recent position paper of the Italian Society of Hypertension [25].

Our results confirm the safety and efficacy of the procedure in terms of systolic BP reduction in such patients, and what is even more relevant, adds information with regard to a scarcely investigated setting such as the application of RSD outside the context of controlled, sponsored trials, revealing the true applicability of RSD in real-life patients with multiple comorbidities and high cardiovascular risk.

Despite the relatively small number of patients (*n* = 40), this is the largest series of RSD cases performed in Italy to date.

A significant and sustained reduction in 24 h systolic BP was observed: − 9.7 mmHg, − 8.4 mmHg and − 11.3 mmHg at 3, 6, and 12 month follow-up after RSD, respectively; as well as a significant reduction in office systolic BP by − 13.9 mmHg and − 19.7 mmHg at 6 and 12 month follow up, respectively. A trend towards an important reduction in diastolic blood pressure was also observed, although it did not reach statistical significance, most likely due to the small sample size and the inclusion of patients with ISH with normal diastolic BP at baseline. Moreover, our data show a reduction in p-PP at each time point that, in previous studies, has been associated with an important reduction in the global cardiovascular risk [26].

All these observations are in line with the results obtained in the pivotal studies conducted on radiofrequency-based RSD systems. Our data confirm and extend these findings by demonstrating significant BP reductions in a population with an eGFR below 45 mL/min/1.73 m^2^, that has previously been excluded from the clinical trials. In our experience the efficacy of RSD in this subgroup is comparable to that in patients with better preserved eGFR. Notably, one patient in our cohort had unilateral nephrectomy prior to RSD and two patients underwent renal transplant without nephrectomy following RSD. Interestingly, all three of these patients experienced BP reductions following RSD.

(Relatively) few previous studies have quantified the safety and efficacy of RSD in CKD patients. This is unfortunate since the association between hypertension and CKD is strong. Hering and colleagues reported good results in a small cohort of 15 cases with uncontrolled resistant hypertension and moderate to severe CKD (baseline eGFR 31 mL/min/1.73 m^2^), with a significant systolic and diastolic OBP reduction (at 3, 6, and 12 months − 25/11, − 32/15, and − 33/19 mmHg, respectively), with no significant effect on ABPM values. [27]. Likewise, Kiuchi and colleagues reported an improvement in renal function in a group of 30 subjects with CKD (baseline eGFR 61.9 ± 23.9) following radiofrequency-based RSD, with a significant decrease in BP values at both OBP and ABPM measurements [28]. Additionally, two meta-analyses of previously published RSD trials showed very little reduction in eGFR following RSD [29] or when compared to sham control [30]. Finally, a recent analysis of the full GSR cohort also showed minimal decline in eGFR (out to) 3 years in RSD patients with eGFR < 60 at baseline, with a significant reduction in 24H systolic BP at 3 years (− 10.1 ± 20.3 mmHg) [31]. Therefore, our results further support the hypothesis that RSD is safe and effective in high risk patients with CKD and persistent hypertension and ESRD. Prospective trials in the population of patients with severe CKD are warranted.

The safety of the RSD procedure has been confirmed in our experience as well, despite the inclusion of patients with multiple comorbidities and high cardiovascular risk profiles. No major complications occurred and kidney function remained stable for up to 12 months after the procedure, with no significant difference in terms of creatinine variation between groups according to baseline eGFR. Our data are in accordance with the 3-year results of the GSR which showed that eGFR decline remained within the expected range in 468 patients who had CKD at inclusion [32]. To guarantee the safety of the procedure in patients with CKD, it is crucial to limit the volume of injected contrast medium. In our series the mean contrast volume was 71.85 ± 39.5 mL, that is significantly lower compared to the data from clinical trials [16, 17].

Although the Spyral HTN trials demonstrated the efficacy of the procedure, one of the main limitations to a broader adoption of RSD is the lack of data in daily clinical practice, the predictability of response, and some safety concerns in high-risk patients. In our modest experience, higher 24 h systolic BP and combined hypertension were found to be predictors of response at univariate analysis, in accordance with previous studies [33]. Moreover, we found that a lower *E*/*E*′ ratio predicts response to RSD. To the best of our knowledge, the predictive value of preserved diastolic function has not been previously described, and may be a marker of an early and reversible stage of hypertension without target organ damage. This observation is hypothesis-generating and deserves larger investigation.

To conclude, no significant difference in safety and efficacy was reported between the two different types of catheter (Flex vs Spyral). This is in line with data from the world’s largest ongoing registry in this setting, the Global Simplicity Registry. Potential differences might be related to additional variables such as increased knowledge regarding renal nerve distribution, the better anatomic approach provided by a distal rather than a proximal application of radiofrequency, and the large number of ablations obtained with the new tetrapolar system. In our case, the possible effect of a procedural learning curve cannot be excluded since the monopolar catheter was used in the first six RSD cases.

We acknowledge several limitations of our analysis. This is a single-center analysis, with a relatively small number of patients, and it was developed during the learning curve of the technique. Investigators were unblinded to the treatment, and there was no control group. Despite our best efforts, follow-up was not feasible in a number of patients in the cohort, mostly due to geographical reasons. Moreover, baseline non-adherence to medical therapy may represent a potential limitation as a possible cause of “pseudo-resistant” hypertension. During follow up, medical adherence was assessed by direct questioning. In conclusion, due to the relatively small sample of patients, potential predictors of the efficacy of RSD has to be read as hypothesis-generating and deserves further analysis in a larger population.

## Conclusion

In a “real-life population” of patients with uncontrolled-resistant hypertension and multiple comorbidities including CKD, RSD is a safe and feasible strategy when applied on top of medical therapy. In our experience RSD resulted in a significant reduction in systolic BP pressure for up to 12 months after the procedure and a trend towards a reduction in diastolic BP.

Future prospective trials should explore the safety and efficacy of RSD in advanced CKD and ESRD.

## Supplementary Information

Below is the link to the electronic supplementary material.

Supplementary file1 (DOCX 17 KB)
