# Supplementary material for: Insights on safety and efficacy of renal artery denervation for uncontrolled-resistant hypertension in a high risk population with chronic kidney disease: first Italian real-world experience
Source: J Nephrol. 2021 Jan 22;34(5):1445–55. doi: 10.1007/s40620-021-00966-7 (PMC8494706; doi:10.1007/s40620-021-00966-7)
Supplement: Supplementary file 1 — Supplementary file1 (DOCX 17 KB) [file 40620_2021_966_MOESM1_ESM.docx]

**Supplementary material:**

**Table S.1**: Procedural and follow-up comparison between the Flex and the Spyral group.

| **BASELINE** | |  | **Overall**  **N=40 (100%)** | **Flex™**  **N=6 (15%)** | **Spyral™**  **N=34 (85%)** | **p-value** |
| --- | --- | --- | --- | --- | --- | --- |
| **Procedural details** | Ablations points | | 36.2 ± 16.0 | 11.33 ± 3.45 | 40.56 ± 13.03 | **p<0.001** |
|  | Main vessel treatment only | | 6; 15% | 6; 15% | 0 | **p<0.001** |
|  | Main vessel + any branch treatment | | 34; 85% | 0 | 34; 85% | **p<0.001** |
|  | Treatment time (min) | | 53 ± 14 | 61.33 ± 16.86 | 51.03 ± 12.76 | p=0.203 |
|  | Radioscopy duration (min) | | 12 ± 5 | 11.17± 6.05 | 12.45± 4.45 | p=0.636 |
|  | Contrast volume (mL) | | 71.85 ± 39.5 | 133.33 ± 69.47 | 61.00 ± 17.79 | **p<0.001** |
|  | Major complication | | 0 | 0 | 0 | - |
|  | Minor complication | | 6; 15% | 1; 16.7% | 5; 14.7% | p=1.000 |
|  | Transient increment of creatinine | | 4; 10% | 1; 16.7% | 3; 8.8% | p=0.493 |
|  | Femoral Pseudoaneurysm | | 2; 5% | 0 | 2; 5.9% | p=1.000 |

**Table S.2**: Office BP change at follow-up between the Flex and the Spyral group.

| Follow-up | Office BP change from baseline  (mmHg) | FLEX™  N=6 (15%) | | SPYRAL™  N=34 (85%) | | P-VALUE |
| --- | --- | --- | --- | --- | --- | --- |
| 3-month | Systolic change (mmHg) | n=6 | -1.5 ±21.44 | n=27 | -9.89 ±27.47 | *p=0.433* |
|  | Diastolic change (mmHg) |  | -3.17 ±7.81 |  | -1.15 ±12.63 | *p=0.624* |
| 6-month | Systolic change (mmHg) | n=6 | -1.33 ±36.69 | n=23 | -15.22 ±23.66 | *p=0.412* |
|  | Diastolic change (mmHg) |  | -12 ±15.85 |  | -2.17 ±14.95 | *p=0.211* |
| 12-month | Systolic change (mmHg) | n=6 | -6 ±19.97 | n=19 | -24 ±28.06 | *p=0.109* |
|  | Diastolic change (mmHg) |  | -3 ±10.64 |  | -4.11 ±18.37 | *p=0.858* |
